# Effects of Transcranial Direct Current Stimulation of Primary Motor Cortex on Reaction Time and Tapping Performance: A Comparison Between Athletes and Non-athletes

**DOI:** 10.3389/fnhum.2019.00103

**Published:** 2019-04-05

**Authors:** Oliver Seidel, Patrick Ragert

**Affiliations:** ^1^Institute for General Kinesiology and Exercise Science, Faculty of Sport Science, University of Leipzig, Leipzig, Germany; ^2^Department of Neurology, Max Planck Institute for Human Cognitive and Brain Sciences, Leipzig, Germany

**Keywords:** tDCS, reaction time, tapping, primary motor cortex, athletes

## Abstract

Recent studies provided compelling evidence that physical activity leads to specific changes on a functional and structural level of brain organization. The observed neural adaptions are specific to the sport and manifested in those brain regions which are associated with neuronal processing of sport-specific skills. Techniques of non-invasive brain stimulation have been shown to induce neuroplastic changes and thereby also facilitate task performance. In the present study, we investigated the influence of transcranial direct current stimulation (tDCS) over the leg area of the primary motor cortex (M1) on simple reaction time tasks (RTT) and tapping tasks (TT) as a comparison between trained football (FB) and handball players (HB) and non-athletes (NA). We hypothesized that anodal tDCS over M1 (leg area) would lead to specific behavioral gains in RTT and TT performance of the lower extremity as compared to sham condition. On an exploratory level, we aimed at revealing if trained athletes would show stronger tDCS-induced behavioral gains as compared to NA, and, furthermore, if there are any differential effects between FB and HB. A total number of 46 participants were enrolled in a sham-controlled, double-blinded, cross-over study. A test block consisting of RTT and TT was performed before, during, after as well as 30 min after a 20-min tDCS application. Additionally, the specificity of tDCS-induced changes was examined by testing upper extremity using the same experimental design as a control condition. Our data showed no group- or sport-specific tDCS-induced effects (online and offline) on RTT and TT neither for lower nor upper extremities. These findings indicate that neither athletes nor NA seems to benefit from a brief period of tDCS application in speed-related motor tasks. However, more knowledge on neuronal processing of RTT and TT performance in trained athletes, the influence of tDCS parameters including stimulation sites, and the effect of inter-individual differences are required in order to draw a comprehensive picture of whether tDCS can help to enhance motor abilities on a high-performance level.

## Introduction

A variety of studies suggest that physical exercise leads to specific changes on a functional and structural level of brain organization (Colcombe et al., 2006; Bullitt et al., 2009; Voss et al., 2010; Erickson et al., 2012). In addition, it has been shown that this neuroplasticity seems to be specific to the individual exercise regime or sport (Jäncke et al., 2009; Park et al., 2009; Schlaffke et al., 2014). On a functional level, the findings of Lulic et al. (2017), using transcranial magnetic stimulation (TMS), indicate that the propensity for exercise-induced functional plasticity is different in high vs. low physically active individuals. In this study, a single session of moderate intensity aerobic exercise increased the amplitude of corticospinal output in the HIGH (physically active) group, and, in contrast, did not alter corticospinal output in the LOW (physically active) group. Apart from the physical activity itself, also the exercise regime leads to specific brain alterations and influences the amount of structural plasticity (Schlaffke et al., 2014). Concerning brain structure, a study by Meier et al. (2016), for example, showed that handball players have an increased volume of gray matter (GM) in the hand area of the primary motor cortex (M1), while ballet dancers are characterized by an increased GM volume in the foot area of M1. These results indicate that the observed functional and structural adaptions are sport-specific/ physical activity-dependent and seem to manifest in those brain regions that are involved in the neural processing of sport-specific skills.

It is well known that M1 is a key region involved in motor control and functions in terms of precision, speed, strength, endurance and execution of daily motor tasks (Levasseur-Moreau et al., 2013). One opportunity to explore the function of certain brain areas can be found in non-invasive brain stimulation methods such as transcranial magnetic (TMS) and/or direct current stimulation (tDCS). To investigate the role of motor-related brain regions during the execution of motor tasks, tDCS is a common method to modulate brain function specifically and thereby induce a possible behavioral change.

tDCS is a non-invasive method for modulating the excitability of certain brain regions by applying a weak direct current to the scalp. It has been proposed that tDCS modulates neural firing rates during stimulation and synaptic strength following long-term stimulation (Stagg and Nitsche, 2011). Using this method, either an increase (by means of anodal tDCS) or a reduction (by means of cathodal tDCS) of the area-specific excitability is possible (Nitsche and Paulus, 2000), as demonstrated by changes in the motor evoked potential (MEP) elicited *via* TMS.

Although tDCS has been mainly used for patients with neurologic disorders (Flöel, 2014; Lattari et al., 2017b) and psychiatric disorders (Aparício et al., 2016), it has also been highlighted as a valuable tool to enhance physical performance in healthy individuals. Current reviews including studies investigating healthy adults provided evidence that anodal tDCS over motor-related brain regions can lead to positive behavioral effects (Banissy and Muggleton, 2013; Machado et al., 2018). For example, a tDCS-induced increase of isometric muscle force has been found in both lower (Tanaka et al., 2009) and upper extremities (Boggio et al., 2006; Hummel et al., 2006; Stagg et al., 2011; Salimpour and Shadmehr, 2014). Further studies have shown that endurance performance (Angius et al., 2018) and both static and dynamic balance regulation (Dutta et al., 2014; Kaminski et al., 2016) can be improved by anodal tDCS. However, concerning anodal tDCS effects in speed-related motor tasks, the current literature is inconsistent. Positive effects have been demonstrated especially in serial and choice reaction time tasks (RTT) with upper extremities (Nitsche et al., 2003b; Verissimo et al., 2016; Drummond et al., 2017; Hupfeld et al., 2017). In studies using simple RTT, the findings are rather contradictory, since both improved reaction times (Carlsen et al., 2015; Devanathan and Madhavan, 2016; Hupfeld et al., 2017) and no effects (Tanaka et al., 2009; Stagg et al., 2011; Horvath et al., 2016) are reported. Only a small number of studies investigated the influence of anodal tDCS on tapping tasks (TT), focusing mainly on (serial) finger TT. The results showed either positive (Tecchio et al., 2010; Saimpont et al., 2016) or null effects (Boehringer et al., 2013), while one reported a significant impairment following anodal tDCS (Stagg et al., 2011). However, concerning tDCS effects on frequency-oriented hand or even foot TT, there is a clear lack of evidence in the current literature.

More recently, there has been great interest in the use of tDCS to enhance sports performance (Davis, 2013; Reardon, 2016) and to facilitate neuroplasticity and training adaptations (Bolognini et al., 2009) in athletes. First, approaches can be found in recent studies showing a tDCS-induced increase of isometric strength of shoulder rotators muscles in handball players (Hazime et al., 2017) and an increased isometric quadriceps strength after stimulation in soccer players (Vargas et al., 2018). Similar results were found by Lattari et al. (2017a) examining tDCS-induced effects on muscle power in individuals with advanced resistance training experience. Furthermore, anodal tDCS is capable to have positive effects on the time of exhaustion in trained individuals performing a cycling task (Vitor-Costa et al., 2015). Beyond that, Okano et al. (2015) studied the effects of 20 min of tDCS with the anode over the left temporal cortex on trained cyclists during an incremental cycling test and found significantly improved peak power, as well as reduced heart rate and perception of effort at submaximal workloads. These findings suggest that tDCS can potentially facilitate the athlete’s performance under laboratory conditions. However, there is no evidence that this could lead to positive transfer effects under field conditions or even during competition. Further risks, opportunities and potential approaches concerning the use of tDCS at an elite sports level have already been discussed by Banissy and Muggleton (2013) and Edwards et al. (2017). It seems clear that more research is needed to clarify the usefulness of tDCS in highly trained individuals (Colzato et al., 2017; Edwards et al., 2017). As maximum performance in fine motor control could not be further improved in elite pianists (Furuya et al., 2013), it needs to be investigated whether similar ceiling effects might apply to the performance of elite athletes as well.

The primary aim of the present study was to investigate the influence of a 20-min anodal tDCS over leg area of the M1 on the performance of trained athletes in simple speed-related motor tasks, using simple reaction time and TT for both upper and lower extremities. According to a systematic review and meta-analysis by Machado et al. (2018) that assessed the effect of tDCS on exercise performance enhancement in healthy adults, no effect was found for cathodal tDCS for any tasks (performance in isometric, isokinetic or dynamic strength exercise and whole-body exercise). Similarly, Tanaka et al. (2009) found no effect of cathodal tDCS over M1 leg area on simple RTT for lower extremities. Hence, the application of cathodal tDCS was waived in the present study. Therefore, the focus was on the question of whether athletes would show stronger anodal tDCS-induced performance gains compared to non-athletes and if sport-specific differences could be determined. In general, we first hypothesized that anodal tDCS over M1 (leg area) would lead to specific behavioral gains in simple reaction time and tapping performance of the foot (not hand, since this can be considered as a kind of control condition) as compared to sham condition (in accordance with Devanathan and Madhavan, 2016; Saimpont et al., 2016). To demonstrate that potential performance gains are in fact related to tDCS, we tested a control group (CG) of participants performing exactly the same procedure but without brain stimulation. Concerning athletes, we expected football and handball players to show superior initial performances as compared to non-athletes. This hypothesis is based on previous studies showing better initial performances in athletes in several motor abilities (Verburgh et al., 2016; Seidel et al., 2017). On an exploratory level, we aimed at revealing if athletes would show stronger tDCS-induced behavioral gains as compared to non-athletes, and, furthermore, if there are any differential effects between football and handball players. Since there is barely evidence concerning tDCS-effects at an elite sports level in speed-related motor tasks, we cannot make direct inferences about the directionality of tDCS-induced behavioral effects.

## Materials and Methods

### Ethics Statement

The study was approved by the local ethics-committee of the Medical Faculty at the University of Leipzig. All participants gave written informed consent to participate in the experiments according to the Declaration of Helsinki, and were compensated for participation.

### Participants

In the present study, a total of 46 healthy, young adults were recruited, divided into three groups of football players (FB), handball players (HB) and non-athletes (NA). To exclude the presence of any neurological disease and/or contraindications, all participants underwent a detailed neurological examination prior to the testing phase. Inclusion criteria for FB and HB consisted of an individual training history of at least 2 years as well as regular practice and regular participation in competitions/matches in their respective sports discipline. NA were not allowed to do more than 2 h of combined sports activities per week. The investigated sample of this study consisted of 13 FB (three females, mean age = 24.00 ± 3.89 years), 12 HB (five females, mean age = 22.50 ± 4.32 years) and 21 NA (11 females, mean age = 26.95 ± 3.43 years). On average, FB trained for 16.31 ± 5.02 years and currently 5.65 ± 2.15 h/week, whereas HB trained for 13.17 ± 4.49 years and currently 8.54 ± 3.84 h/week in their respective sports disciplines. On the other hand, NA performed an average of less than 2 h of combined sports activities per week (1.41 ± 1.32 h/week). Additionally, all participants (FB, HB and NA) with regular practice of musical instruments were excluded from participation in this study. This was motivated by the fact that recent studies have shown that musical training induces functional and structural plasticity in motor-related brain regions (Steele et al., 2013; Vollmann et al., 2014) which in turn might affect the amount of tDCS effect. As assessed by the Edinburgh Handedness Inventory (Oldfield, 1971), all participants were right-handed [mean laterality quotient (LQ) of FB: 84.02 ± 16.45; HB: 95.83 ± 8.14; NA: 90.15 ± 14.15].

Furthermore, a CG was tested to ensure that potential behavioral changes in simple reaction time and tapping frequency are in fact tDCS related and not a mere effect of fatigue or learning. A total of six male and six female right-handed (mean LQ: 80.4 ± 17.1) participants (*n* = 12) in this group with an mean age of 21.25 ± 1.14 years and sports-related activities of 4.85 ± 3.86 h/week had to perform the whole procedure (see “Experimental Design” section for further details) but without tDCS.

### Experimental Design

**Figure 1:**
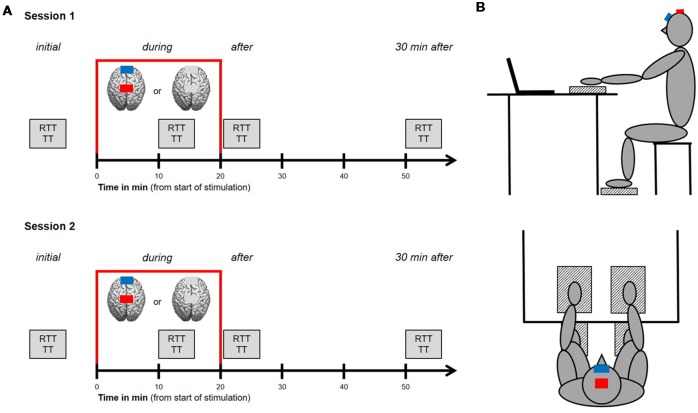
Study design and experimental setup. **(A)** Procedures for session 1 and 2. Study procedure for both sessions was identical, starting with an initial run of a test block consisting of reaction time tasks (RTT) and tapping tasks (TT) for upper and lower extremities. Afterwards, transcranial direct current stimulation (tDCS) was applied over the leg area of the primary motor cortex (M1 leg area) for a period of 20 min (indicated by the red frame). Participants received either the anodal tDCS condition or the control condition, where sham tDCS was applied. Another run of the aforementioned test block was performed after 10 min of stimulation (during tDCS, online) as well as directly after and 30 min after stimulation has ended (offline). **(B)** Experimental setup. Participants were seated 1 m away from a computer monitor, upright on a stool (hips and knees at 90°) with their hands resting on and their feet resting under a table in front of them. Next to each hand and foot, with a defined distance of 10 cm, a custom made force plate (indicated as patterned boxes) was installed. Indicated by the red (anode) and blue (cathode) boxes, the anode was placed over the M1 leg area target region, the cathode (reference electrode) was placed over the middle of the forehead.

A sham-controlled, double-blinded, cross-over design was carried out. The study was compromised of two sessions that were separated by at least 24 h in order to avoid task-related impacts of cognitive or muscular fatigue. Study procedure for both sessions was identical (see Figure 1A), starting with an initial run of a test block consisting of RTT and TT for upper and lower extremities (see “Motor Tasks” section for further details). Afterward, tDCS was applied over the leg area of the (M1 leg area) for a period of 20 min. Participants received either the anodal tDCS condition or the control condition, where sham tDCS was applied [see “Transcranial Direct Current Stimulation (tDCS)” section for further details]. For each participant, the type of stimulation was randomly assigned to either session 1 or 2. Another run of the aforementioned test block was performed after 10 min of stimulation (during tDCS, online) as well as directly after and 30 min after stimulation has ended (offline). The second test block was performed after 10 min of stimulation because previous studies have demonstrated that a time of 9–13 min is required to obtain an increase in cortical excitability for up to 1.5 h (Nitsche and Paulus, 2000). Participants were instructed to avoid alcohol and caffeine 24 h prior to each session because of their well-known influences on motor control and central nervous system (CNS) functioning (Pesta et al., 2013). Additionally, participants were asked to report their daily activities 48 h before both sessions, their current levels of attention, fatigue and discomfort on a visual analog scale (pre and post), as well as their individual amount of sleep the night before the experimental sessions, to sufficiently control for this matter.

### Transcranial Direct Current Stimulation (tDCS)

For tDCS, a weak direct current of 2 mA was delivered for 20 min by means of two surface electrodes using a battery driven-stimulator (neuroConn GmbH, Ilmenau, Germany). For each session, either anodal tDCS or sham tDCS was applied to the bilateral M1 leg area. While the anode (7 cm × 5 cm) was placed over the M1 leg area target region, the cathode (10 cm × 10 cm, reference electrode) was placed over the middle of the forehead. The anatomical landmark for M1 leg area was chosen according to the 10–20 system and the anode was placed over the vertex (Cz) on the mid-sagittal line (Madhavan and Stinear, 2010; Laczó et al., 2014). Cz was determined over the intersection of the courses nasion to inion and left preauricular point to right preauricular point according to Jurcak et al. (2007). tDCS was applied using two saline-soaked (0.9% NaCl) sponges and flexible elastic straps were used to fixate the electrodes on the head. The current was ramped up for 30 s at the beginning of tDCS eliciting a transient tingling sensation on the scalp that faded over seconds (Nitsche et al., 2003a; Gandiga et al., 2006) and also ramped down for 30 s. During sham tDCS, the current was increased, maintained and decreased for 30 s each. According to Gandiga et al. (2006), this is enough time to identify the presence of the current with no effective brain stimulation. The electrical resistance was constantly monitored on the stimulator’s display within a range between 5 and 10 kΩ. The adverse effects were evaluated after each application through spontaneous reports of any unpleasant sensations such as burning, tingling, headache or nausea.

### Motor Tasks

During each session, participants were seated 1 m away from a computer monitor, upright on a stool (hips and knees at 90°) with their hands resting on and their feet resting under a table in front of them (see Figure 1B). Next to each hand and foot, with a defined distance of 10 cm, a custom made force plate was installed. Participants were instructed to rest and relax their inactive extremities in this position. Facing the computer monitor, participants performed four runs of a test block (initial, during, after and 30 min after tDCS) consisting of speed-related motor tasks. Each block consisted of two runs of a simple RTT and two runs of a TT for each hand and foot separately. Therefore, a total amount of eight RTTs and eight TTs had to be performed with a total duration of approx. 8 min. The order of these tasks was randomized for each block and the software avoided two or more tasks for the same extremity in a row. Between each task was a short rest period of 3 s when the upcoming task appeared on the computer monitor.

#### Simple Reaction Time Task (RTT)

For simple RTT, participants had to place their active hand (respectively foot) at the defined spot 10 cm away from the respective force plate. In this position, they were instructed to face the computer monitor and read the upcoming task carefully. After a countdown of 3 s, indicating the start of the run, participants were asked to press the respective force plate as quickly as possible in response to the appearance of a visual stimulus (cross) on the computer monitor. During one RTT, a total of 15 trials had to be responded in this manner with a randomized inter-trial interval of 0.5–2.0 s to avoid anticipation of trial onsets. Between each trial, the active hand (respectively foot) had to be placed back to the defined spot. For each trial, the time interval (ms) between the onset of the trial (cross) and the response was recorded as an outcome measure.

#### Tapping Task (TT)

For TT, participants were asked to take the same position as previously described for RTT. After a countdown of 3 s, participants started the run on their own with their first touch of the respective force plate. Subsequently, they had to press the force plate as often as possible over a period of 20 s. Concerning upper extremity TT, participants were instructed to tap in the center of the force plate with a flat hand. For the lower extremity counterpart, they were asked to keep the heel up in the air and to tap with their forefoot. As an outcome measure, tapping frequency (Hz) was recorded.

### Analysis

For each test block, two runs of 15 trials were recorded for the left hand (HL), right hand (HR), left foot (FL) and right foot (FR), respectively. Afterward, these 30 reaction times of one block were averaged for each extremity separately. Outliers were defined as values <100 ms and >1,000 ms (Geiger et al., 2018) of each participant and were excluded from the averages. The lower limit was determined since all reaction times <100 ms are considered to be unphysiological and only in very few cases have been measured so far for the much faster auditory reaction times (Pain and Hibbs, 2007). After averaging all valid reaction times, this resulted in one value for RTT before (*initial*), *during*, *after* and 30 min *after* tDCS stimulation. Baseline differences were tested using a univariate ANOVA and revealed significant differences between groups (see “Results” section for further details). Hence, all values were normalized to *initial* (= 100%).

Concerning TT, two runs of 20 s were recorded for each test block for HL, HR, FL and FR, respectively. First, the total amount of taps during one run resulted in an average tapping frequency over 20 s that was averaged for both runs for each extremity (TT_20_). Second, the total amount of taps during the first 3 s was considered, extracting the tapping frequency of the fastest second (TT_max_). According to RTT, this resulted in one value for TT_20_ and TT_max_ before (*initial*), *during*, *after* and 30 min *after* tDCS stimulation. Due to baseline differences (see “Results” section for further details), values were also normalized to *initial* (= 100%).

All statistical analyses were performed with the software SPSS 22 (IBM, Armonk, NY, USA) using parametric tests since Shapiro-Wilk test revealed that RTT and TT data were normally distributed. As already described above, baseline differences were examined using an univariate ANOVA with factor group (FB vs. HB vs. NA) using Gabriel and Games-Howell *post hoc* tests, respectively to analyze the differences if necessary. A 2 × 3 × 3 repeated measures ANOVA was conducted to analyze the mean normalized values of RTT, TT_max_ and TT_20_ of each group and each extremity for three test blocks of the tasks (first within-subject factor), including stimulation condition (anodal vs. sham) as second within-subject factor and group (FB vs. HB vs. NA) as between-subject factor. Regarding the first within-subject factor, *initial* was not included since data were normalized and level *initial* would not have any variance across participants since all of them would have a value of 100%.

For the CG (without tDCS), a repeated measures ANOVA with factor test block (within-subjects factor) was conducted. Additionally, we computed the test-retest reliability using an intraclass correlation coefficient (ICC) to examine whether potential performance gains are in fact tDCS related or an effect of fatigue or learning.

When the respective interactions were significant, also Gabriel and Games-Howell *post hoc* tests, respectively were applied to analyze the differences. The critical level of significance for RTT and TT differences in all tests was set to *p* < 0.05 and Bonferroni adjusted for multiple comparisons. If necessary, data were corrected for sphericity using Greenhouse-Geisser correction. Partial eta-squared ($\eta_{\text{p}}^{2}$) for ANOVAs are provided as measures of effect size and used to aid in the interpretation of inferential statistics. As a rule of thumb, introduced by Miles and Shevlin (2000), $\eta_{\text{p}}^{2}$ ≥ 0.01 is considered to be a small, $\eta_{\text{p}}^{2}$ ≥ 0.06 a medium, and $\eta_{\text{p}}^{2}$ ≥ 0.14 a large effect. Additionally, as recommended for tDCS studies by Biel and Friedrich (2018), Bayes factors (BF), a useful tool for evaluating evidence both for the research hypothesis and for the null hypothesis (Dienes, 2011; Kruschke, 2011), are reported for repeated measures ANOVAs using JASP (Jeffreys’s Amazing Statistics Program, Marsman and Wagenmakers, 2017). BFs above 1 indicate evidence for H1 over H0, whereas BFs below 1 suggest the exact opposite. If BFs are above 3 or below 0.33, the strength of evidence for one hypothesis compared to its competing hypothesis is regarded as noteworthy (Jeffreys, 1961; Lee and Wagenmakers, 2013). Thus, BFs between 0.33 and 3 are considered as inconclusive, or only anecdotal evidence for any hypothesis.

## Results

### Test-Retest Reliability of RTT and TT

To exclude that the pure repetition of RTT and TT would lead to significant behavioral alterations, we performed a test-retest analysis using a CG (*n* = 12). We found no statistically significant alterations neither in RTT performance (rmANOVA, main effect of test block, HL: *F*_(3,33)_ = 0.363, *p* = 0.780, $\eta_{\text{p}}^{2}$ = 0.032; HR: *F*_(3,33)_ = 0.215, *p* = 0.886, $\eta_{\text{p}}^{2}$ = 0.019; FL: *F*_(2.139,23.531)_ = 2.002, *p* = 0.155, $\eta_{\text{p}}^{2}$ = 0.154; FR: *F*_(3,33)_ = 0.290, *p* = 0.832, $\eta_{\text{p}}^{2}$ = 0.026) nor in TT performance (rmANOVA, main effect of test block, HL: *F*_(1.914,21.055)_ = 2.227, *p* = 0.134, $\eta_{\text{p}}^{2}$ = 0.168; HR: *F*_(1.379,15.172)_ = 2.622, *p* = 0.118, $\eta_{\text{p}}^{2}$ = 0.192; FL: *F*_(1.600,17.598)_ = 1.695, *p* = 0.214, $\eta_{\text{p}}^{2}$ = 0.133; FR: *F*_(1.525,16.770)_ = 2.103, *p* = 0.160, $\eta_{\text{p}}^{2}$ = 0.160) performance. These findings were confirmed by good intrasession reliabilities according to Larsson et al. (1999) for RTT (ICC_HL_ (33,11) = 0.908; ICC_HR_ (33,11) = 0.884; ICC_FL_ (33,11) = 0.845; ICC_FR_ (33,11) = 0.897) and TT (ICC_HL_ (33,11) = 0.974; ICC_HR_ (33,11) = 0.952; ICC_FL_ (33,11) = 0.947; ICC_FR_ (33,11) = 0.940).

### Initial Group Comparisons of RTT and TT

#### Initial RTT Performance

**Figure 2:**
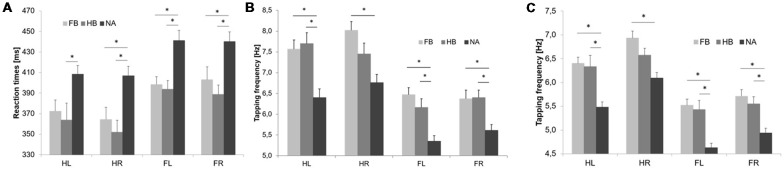
Initial RTT and TT results. Values are mean ± SE of left hand (HL), right hand (HR), left foot (FL) and right foot (FR), respectively. Light gray bars represent football players (FB), medium gray bars represent handball players (HB) and dark gray bars represent non-athletes (NA). *(*p* < 0.05) indicates significant differences between groups in their initial performances. Initial simple RTT values **(A)**, TT_max_ values **(B)** and TT_20_ values **(C)** differed significantly between groups indicating superior performances in FB and HB as compared to NA.

Initial RTT values differed significantly between groups indicating superior RTT performances in FB and HB as compared to NA (see Figure 2A). uANOVA revealed a significant main effect of group in HL (*F*_(2,43)_ = 4.752, *p* = 0.014, $\eta_{\text{p}}^{2}$ = 0.181), HR (*F*_(2,43)_ = 7.910, *p* = 0.001, $\eta_{\text{p}}^{2}$ = 0.269), FL (*F*_(2,43)_ = 9.272, *p* = 0.000, $\eta_{\text{p}}^{2}$ = 0.301) and FR (*F*_(2,43)_ = 6.863, *p* = 0.003, $\eta_{\text{p}}^{2}$ = 0.242). *Post hoc* analyses showed significant differences between FB and NA in HR (*p*_adjusted_ = 0.018), FL (*p*_adjusted_ = 0.004) and FR (*p*_adjusted_ = 0.040) as well as between HB and NA in HL (*p*_adjusted_ = 0.023), HR (*p*_adjusted_ = 0.002), FL (*p*_adjusted_ = 0.002) and FR (*p*_adjusted_ = 0.003). However, there were no significant differences between FB and HB (HL: *p*_adjusted_ = 0.950; HR: *p*_adjusted_ = 0.839; FL: *p*_adjusted_ = 0.983; FR: *p*_adjusted_ = 0.770).

#### Initial TT_max_ Performance

Initial maximum tapping frequency (TT_max_) differed significantly between groups indicating superior TT_max_ performances in FB and HB as compared to NA (see Figure 2B). uANOVA revealed a significant main effect of group in HL (*F*_(2,43)_ = 10.729, *p* = 0.000, $\eta_{\text{p}}^{2}$ = 0.333), HR (*F*_(2,43)_ = 8.525, *p* = 0.001, $\eta_{\text{p}}^{2}$ = 0.284), FL (*F*_(2,43)_ = 14.231, *p* = 0.000, $\eta_{\text{p}}^{2}$ = 0.398) and FR (*F*_(2,43)_ = 7.501, *p* = 0.002, $\eta_{\text{p}}^{2}$ = 0.259). *Post hoc* analyses showed significant differences between FB and NA in HL (*p*_adjusted_ = 0.002), HR (*p*_adjusted_ = 0.001), FL (*p*_adjusted_ = 0.000) and FR (*p*_adjusted_ = 0.008) as well as between HB and NA in HL (*p*_adjusted_ = 0.001), FL (*p*_adjusted_ = 0.003) and FR (*p*_adjusted_ = 0.006). However, there were no significant differences between FB and HB (HL: *p*_adjusted_ = 0.973; HR: *p*_adjusted_ = 0.292; FL: *p*_adjusted_ = 0.536; FR: *p*_adjusted_ = 0.999).

#### Initial TT_20_ Performance

Initial average tapping frequency over 20 s (TT_20_) differed significantly between groups indicating superior TT_20_ performances in FB and HB as compared to NA (see Figure 2C). uANOVA revealed a significant main effect of group in HL (*F*_(2,43)_ = 13.081, *p* = 0.000, $\eta_{\text{p}}^{2}$ = 0.378), HR (*F*_(2,43)_ = 9.995, *p* = 0.000, $\eta_{\text{p}}^{2}$ = 0.317), FL (*F*_(2,43)_ = 15.682, *p* = 0.000, $\eta_{\text{p}}^{2}$ = 0.422) and FR (*F*_(2,43)_ = 11.426, *p* = 0.000, $\eta_{\text{p}}^{2}$ = 0.347). *Post hoc* analyses showed significant differences between FB and NA in HL (*p*_adjusted_ = 0.000), HR (*p*_adjusted_ = 0.000), FL (*p*_adjusted_ = 0.000) and FR (*p*_adjusted_ = 0.000) as well as between HB and NA in HL (*p*_adjusted_ = 0.001), FL (*p*_adjusted_ = 0.000) and FR (*p*_adjusted_ = 0.004). However, there were no significant differences between FB and HB (HL: *p*_adjusted_ = 0.988; HR: *p*_adjusted_ = 0.282; FL: *p*_adjusted_ = 0.963; FR: *p*_adjusted_ = 0.815).

### tDCS-Induced Effects on RTT and TT Performance

#### tDCS-Induced Effects on RTT Performance

**Figure 3:**
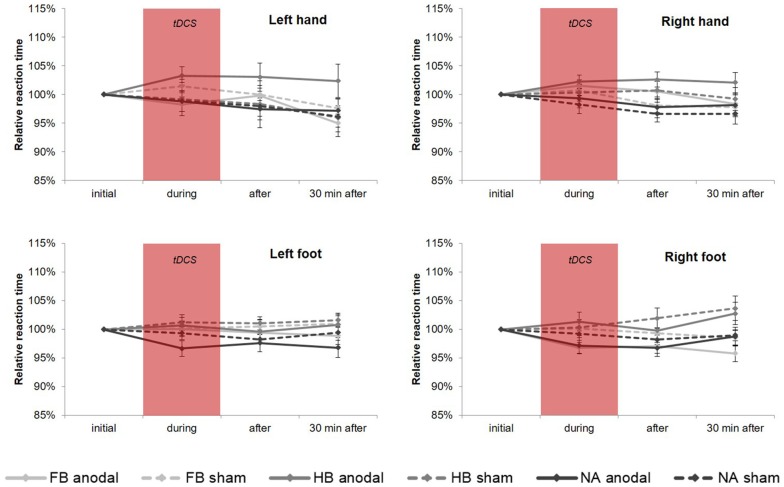
tDCS-induced effects on simple RTT performance. Diagrams include normalized (% of initial values) simple reaction time values (mean ± SE) of left hand (HL), right hand (HR), left foot (FL) and right foot (FR), respectively for before (initial), during, after as well as 30 min after a 20-min tDCS which is indicated by the red box. Light gray lines represent football players (FB), medium gray lines represent handball players (HB) and dark gray lines represent non-athletes (NA). The solid lines define values for anodal tDCS and the corresponding dashed lines indicate values for sham tDCS.

Regarding tDCS-induced effects on RTT performance in the upper extremities (see Figure 3), rmANOVA revealed a non-significant time × group × condition interaction (HL: *F*_(4,86)_ = 0.741, *p* = 0.566, $\eta_{\text{p}}^{2}$ = 0.033, BF = 0.077; HR: *F*_(4,86)_ = 0.321, *p* = 0.863, $\eta_{\text{p}}^{2}$ = 0.015, BF = 0.064). Only factor time showed a significant influence on RTT performance (HL: *F*_(2, 86)_ = 9.228, *p* = 0.000, $\eta_{\text{p}}^{2}$ = 0.177; HR: *F*_(2,86)_ = 4.622, *p* = 0.012, $\eta_{\text{p}}^{2}$ = 0.097). Moreover, *post hoc* tests revealed a significant influence of factor group in HR directly after tDCS (*F*_(2,43)_ = 4.267, *p*_adjusted_ = 0.020, $\eta_{\text{p}}^{2}$ = 0.166).

For FL, rmANOVA examined a significant influence of factor group (*F*_(2, 43)_ = 3.564, *p* = 0.037, $\eta_{\text{p}}^{2}$ = 0.142) indicating significant differences between HB and NA (*p*_adjusted_ = 0.045), although there was no significant time × group × condition interaction (*F*_(3.311, 71.190)_ = 0.788, *p* = 0.516, $\eta_{\text{p}}^{2}$ = 0.035, BF = 0.078). For FR, results showed a significant time × group interaction (*F*_(4, 86)_ = 2.504, *p* = 0.048, $\eta_{\text{p}}^{2}$ = 0.104) and a significant influence of factor group (*F*_(2,43)_ = 4.434, *p* = 0.018, $\eta_{\text{p}}^{2}$ = 0.171) indicating differences between FB vs. HB (*p*_adjusted_ = 0.037) and HB vs. NA (*p*_adjusted_ = 0.029). The highest influence of factor group has been found 30 min after tDCS (*p*_adjusted_ = 0.001). However, there was no significant influence of tDCS condition (time × group × condition: *F*_(4,86)_ = 1.061, *p* = 0.381, $\eta_{\text{p}}^{2}$ = 0.047, BF = 0.088). On a group level, RTT performance in FB differed significantly between anodal and sham (*p*_adjusted_ = 0.022) indicating a tDCS-induced RTT performance gain of 3.21%.

#### tDCS-Induced Effects on TT_max_ Performance

**Figure 4:**
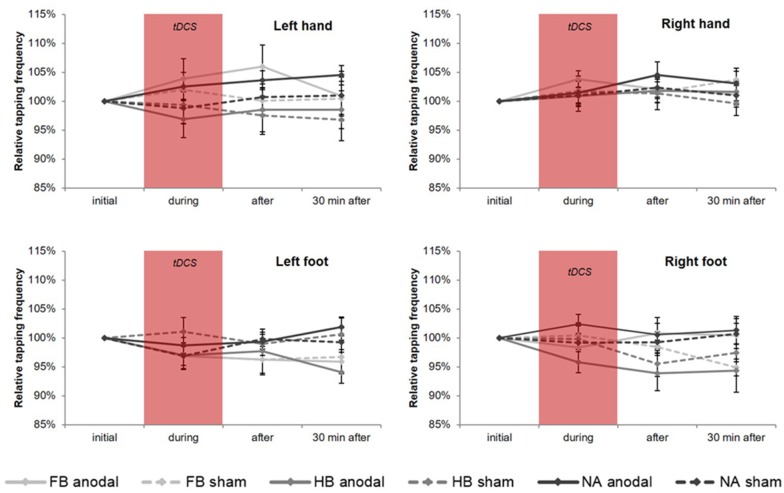
tDCS-induced effects on maximum tapping frequency (TT_max_). Diagrams include normalized (% of initial values) TT_max_ values (mean ± SE) of left hand (HL), right hand (HR), left foot (FL) and right foot (FR), respectively for before (initial), during, after as well as 30 min after a 20-min tDCS which is indicated by the red box. Light gray lines represent football players (FB), medium gray lines represent handball players (HB) and dark gray lines represent non-athletes (NA). The solid lines define values for anodal tDCS and the corresponding dashed lines indicate values for sham tDCS.

rmANOVA revealed no significant time × group × condition interaction for TT_max_ performance (see Figure 4), neither in upper (HL: *F*_(3.272,70.351)_ = 1.114, *p* = 0.352, $\eta_{\text{p}}^{2}$ = 0.049, BF = 0.091; HR: *F*_(4,86)_ = 1.485, *p* = 0.214, $\eta_{\text{p}}^{2}$ = 0.065, BF = 0.123) nor in lower extremities (FL: *F*_(3.334,71.682)_ = 2.039, *p* = 0.110, $\eta_{\text{p}}^{2}$ = 0.087, BF = 0.153; FR: *F*_(3.009,64.689)_ = 1.553, *p* = 0.209, $\eta_{\text{p}}^{2}$ = 0.067, BF = 0.210). The same applies to all *post hoc* tests, which also showed no significant results.

#### tDCS-Induced Effects on TT_20_ Performance

**Figure 5:**
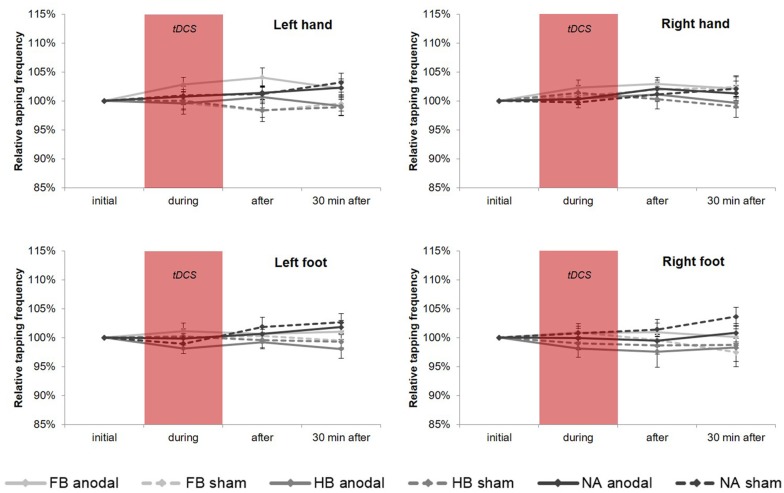
tDCS-induced effects on average tapping frequency over 20 s (TT_20_). Diagrams include normalized (% of initial values) TT_20_ values (mean ± SE) of left hand (HL), right hand (HR), left foot (FL) and right foot (FR), respectively for before (initial), during, after as well as 30 min after a 20-min tDCS which is indicated by the red box. Light gray lines represent football players (FB), medium gray lines represent handball players (HB) and dark gray lines represent non-athletes (NA). The solid lines define values for anodal tDCS and the corresponding dashed lines indicate values for sham tDCS.

Regarding the upper extremities, rmANOVA revealed a non-significant time × group × condition interaction for TT_20_ performance (see Figure 5) in HL (*F*_(4,86)_ = 0.672, *p* = 0.613, $\eta_{\text{p}}^{2}$ = 0.030, BF = 0.070) and HR (*F*_(4,86)_ = 0.945, *p* = 0.442, $\eta_{\text{p}}^{2}$ = 0.042, BF = 0.095). However, findings in HL showed a significant time × condition interaction (*F*_(2,86)_ = 4.540, *p* = 0.013, $\eta_{\text{p}}^{2}$ = 0.095) indicating a significant influence of tDCS condition directly after stimulation (*post hoc* test: *p*_adjusted_ = 0.011). Moreover, subsequent comparisons on group level for HL revealed a significant difference between anodal and sham condition in FB (*p*_adjusted_ = 0.033) indicating a tDCS-induced performance gain of 4.06% in TT_20_.

However, regarding the lower extremities, TT_20_ findings showed no significant results (see also Figure 5) neither in rmANOVA (FL: *F*_(3.090,66.440)_ = 1.019, *p* = 0.392, $\eta_{\text{p}}^{2}$ = 0.045, BF = 0.097; FR: *F*_(3.239,69.633)_ = 1.061, *p* = 0.375, $\eta_{\text{p}}^{2}$ = 0.047, BF = 0.120) nor in all *post hoc* tests.

## Discussion

The present study aimed to investigate whether 20 min of anodal tDCS over the leg area of the M1 is capable to affect motor performance in a simple reaction time (RTT) and tapping task (TT) for both upper and lower extremities. Here, trained athletes of different sports disciplines [football players (FB) and handball players (HB)] were tested to investigate possible tDCS-induced behavioral gains using speed-related motor tasks. The study focused on the question of whether athletes would differ in their behavioral response to tDCS compared to non-athletes (NA) and if sport-specific differences could be determined. In line with previous findings, we revealed no differences between anodal and sham tDCS conditions neither on RTT (Tanaka et al., 2009; Stagg et al., 2011; Horvath et al., 2016) nor on TT (Boehringer et al., 2013) performance. Thus, our results indicate that the application of tDCS over M1 leg area did not elicit performance enhancement neither in athletes nor in NA. Future studies can use this knowledge to identify valid and suitable conditions that could lead to tDCS-induced performance gains on speed-related motor tasks with regard to different sports and other responsible brain regions such as cerebellum.

### Superior Initial Performances in Athletes Compared to Non-athletes

We hypothesized that athletes would show better RTT and TT performances as compared to NA which was confirmed in both tasks. As well known, physical training has a positive effect on both reaction time (Davranche et al., 2006) and speed (Little and Williams, 2005). Since FB and HB usually integrate speed-related tasks for upper and lower extremities into their practice routine, it is reasonable to assume that this translates into superior performance in RTT and TT compared to performance of NA. Even an acute short-term physical exercise is capable to improve motor time in a simple and choice RTT as it has been shown by Davranche et al. (2006) and Kashihara and Nakahara (2005). Furthermore, it has been reported by several studies that the dynamic visual acuity of athletes was superior to that of NA (Ishigaki and Miyao, 1993) and that athletes were faster in RTTs than NA (Yandell and Spirduso, 1981; Ando et al., 2001; Akarsu et al., 2009; Atan and Akyol, 2014; Kuan et al., 2018). These findings can be explained by the fact that hand-eye coordination plays an important role especially in sports that require high motor hand skills such as team sports and racket sports (Paul et al., 2011; Laby et al., 2018). Moreover, this is also reasonable for sports depending on high motor foot skills such as football. In a recent study by Atan and Akyol (2014), a large number of athletes from different sports branches (football, basketball, judo, track and field, taekwondo) performed a simple RTT of left and right hand in comparison to NA. As a conclusion they found that NA’s reaction time parameters were worse than the most branch athletes. In addition to that, reaction time parameters of athletes did not differ between sports branches (except judokas) which could be confirmed by the present study.

### No Effect of tDCS on RTT and TT Performance

We further hypothesized that anodal tDCS over M1 leg area would lead to specific behavioral gains in simple reaction time and tapping performance of the foot as compared to sham condition. Using common tDCS parameters [see “Transcranial Direct Current Stimulation (tDCS)” section for further details], our results showed no effect of anodal tDCS on RTT and TT performance neither as online gains nor offline.

Concerning RTT results, our findings go in line with a previous study by Tanaka et al. (2009). In this cross-over study, a total number of 10 participants performed hand and foot RTTs before and during as well as 10, 30 and 60 min after anodal, cathodal or sham tDCS, respectively. Stimulation was applied for 10 min over the left leg representation of the right motor cortex with an intensity of 2 mA (for anodal and cathodal condition). The authors stated that the anodal tDCS over the leg motor cortex did not change the leg RTT performance contralateral to the stimulation. From their point of view, that might be due to performance ceiling, task sensitivity or stimulation strength and/or duration. With regard to our results, we could show that even doubling the stimulation duration did not lead to a significant enhancement on the behavioral level. Concerning task sensitivity, some studies suggest that tDCS effects depend upon task-difficulty and individual level of task performance (Kwon et al., 2015; Mizuguchi et al., 2018). Hence, the complexity and sensitivity of simple speed-related motor tasks that were used in the present study might be too low to induce a modulatory tDCS effect on a behavioral level. Using a choice RTT as a more complex task, Drummond et al. (2017) were able to demonstrate enhanced choice reaction times in left and right hand after stimulating M1 for 10 min with an intensity of 1 mA. Furthermore, Hupfeld et al. (2017) provide evidence that a choice RTT is more sensitive to benefit from tDCS.

In contrast, the simple reaction time in hand motor tasks has been reported to be facilitated by anodal tDCS (Hummel and Cohen, 2005; Hummel et al., 2006). Tanaka et al. (2009) assume that, because of low spatial focality of tDCS, anodal tDCS in the previous studies stimulated not only the hand motor cortex but also parts of the premotor cortex. Since this specific brain region is responsible for externally triggered movements (Goldberg, 1985; Wessel et al., 1997; Crosson et al., 2001), it would be reasonable to suppose that RTT performance might be facilitated by tDCS over this area. This clearly elucidates that M1 is only one of several brain regions that is eligible to induce behavioral changes by tDCS in a huge variety of motor tasks. Several studies show that also the cerebellum might play an important role in speed-related motor tasks. In a study by Martin et al. (2006), magnetoencephalography (MEG) was used to measure brain activity while participants performed a simple RTT. The cerebellar results may reflect a number of possible factors, including a role in timing, response readiness, prediction and attention. This is confirmed by an investigation by Théoret et al. (2001), showing no effect of repetitive TMS (rTMS) of the lateral cerebellum or motor cortex, and sham stimulation, on performance of a paced-finger-tapping task (PFT) but following a 5 min train of 1 Hz rTMS to the medial cerebellum.

Concerning TT performance, we hypothesized to elucidate tDCS-induced effects at least on tapping frequency over 20 s since this task is mainly influenced by neuromuscular fatigue (Arias et al., 2012). There is compelling evidence that neuromuscular fatigue, that is defined as the exercise-dependent decrease in the ability of muscle fibers to generate force, occurs due to both central and peripheral factors (Gandevia, 2001). In a previous study, Cogiamanian et al. (2007) investigated whether tDCS delivered over motor cortex would have any effect on fatigue in normal volunteers assessing the endurance time for a submaximal isometric contraction of left elbow flexors. Their findings indicate that anodal stimulation had effects consistent with a reduction in fatigue in comparison to both no stimulation and cathodal stimulation. According to Banissy and Muggleton (2013), these results lead to the assumption that it is possible to modulate fatigue to a large degree with tDCS stimulation. Contrarily, in our case, neither tapping performance over 20 s nor maximum tapping frequency were influenced by anodal tDCS. Therefore, it is absolutely essential to reveal the underlying neural mechanisms of maximum fast movements and movements that are influenced by neuromuscular fatigue in order to use possible tDCS benefits in any sports training.

Taken together, we showed that tDCS is not capable of evoking enhanced performance in speed-related motor tasks. As argued above, the outcome of tDCS seems to be affected by multiple factors involving task characteristics and individual determinants (Ridding and Ziemann, 2010). Furthermore, little is known about neuronal correlates of RTT and TT performance. Therefore, more research is needed to draw a comprehensive picture on speed-related motor abilities in healthy adults and how non-invasive brain stimulation techniques may interact with such complex coordinative behavior.

### tDCS-Effects in Athletes

On an exploratory level, we hypothesized that athletes would show stronger tDCS-induced behavioral gains as compared to NA, and, furthermore, if there are any differential effects between FB and HB. This is based on the assumption that tDCS is capable to broadly modulate brain activity, but, as pointed out by Edwards et al. (2017), it remains to be conclusively determined whether it can improve sports performance at an elite level. Our results indicate that athletes also did not benefit from tDCS stimulation on a behavioral level as did NA. This is partly due to the ceiling effect that may have occurred in RTT and TT, but is also due to the high specificity of the brain of trained athletes. According to the “neural efficiency” hypothesis (Dunst et al., 2014), the athlete’s brain works differently when performing a task compared to NA. More precisely, it consumes less neural resources for the same task. Another peculiarity is based on the concept of “homeostatic plasticity” in human subjects, suggesting that homeostatic mechanisms operating across hemispheric boundaries contribute to regulating motor cortical function in the M1 as previously shown by Ragert et al. (2009). In terms of the present study, this means that a high level of performance in a specific task in combination with an external stimulation can lead to a decline in physical performance. Consequently, anodal tDCS over M1 can induce inhibition of cortical excitability or a null effect on a behavioral level in trained athletes.

However, it is not legitimate to claim that tDCS has no effect on trained athletes *per se*. The reviews of Banissy and Muggleton (2013) and Edwards et al. (2017) include a number of studies showing positive tDCS-induced effects on motor abilities like muscle power and endurance in athletes. Nevertheless, Banissy and Muggleton (2013) draw attention to the point that currently much of the evidence supporting this is theoretical, having been obtained from individuals not involved in a high standard of sport. While this does not apply to the present study, the investigation of more homogeneous groups of athletes might also lead to different results. Although the level of FB and HB was high, they differed quite in their individual training history or in their current training effort.

### Study Limitations

In the present study, we used anodal tDCS to induce a possible behavioral change in the performance of athletes and NA in speed-related motor tasks. To get a better understanding of the neuronal correlates of RTT and TT performance and potential tDCS effects on neuronal networks, further studies that combine neurophysiological assessments of brain activation with behavioral outcome measures are needed. Our findings indicate that the target region (M1 leg area) seems to be less responsible for RTT and TT performance in the lower extremities. Therefore, in future studies, the role of other key regions such as cerebellum or supplementary motor area (SMA) needs to be further investigated. Even though we did not detect any tDCS-induced effects on RTT and TT performance in our study population, it has been previously shown that tDCS affects other motor abilities in athletes. Additionally, we did not investigate the role of multiple tDCS-sessions on RTT and TT performance and did not test for any long-term effects. It is worth considering that multiple tDCS application sessions may have induced stronger behavioral effects that could be more persistent. Following up on this, future studies should also address the problem of optimal stimulation duration and intensity. Concerning polarity, the chance to obtain a different result using cathodal tDCS over M1 leg area is very little since previous findings suggest that it is more difficult to suppress the excitability of the leg motor cortex with cathodal tDCS than the hand area of the motor cortex (Jeffery et al., 2007). This might be due to the leg motor cortex having fewer inhibitory circuits than the hand motor cortex, or cathodal current might be less effective in M1 leg area because of the different orientation and position of the leg motor cortex relative to the scalp (Jeffery et al., 2007; Tanaka et al., 2009). However, this study was the first step in understanding the effect of a single tDCS session on the performance in simple speed-related motor tasks in trained athletes. For future studies, it is conceivable that a more sensitive motor task, as well as a homogeneous study group at a high performance level, can nevertheless lead to a positive tDCS-induced effect in athletes.

## Conclusion

Previous research provides evidence that the application of tDCS is capable to affect the performance in various motor abilities. This is not only true for patients or healthy adults, but also for trained athletes who represent a highly specific group of experts regarding their neuronal adaptions on long-term physical activity. The present study contributes to current approaches to increase sports performance using non-invasive stimulation methods. Our results provide novel quantitative evidence that neither athletes nor NA seems to benefit from a brief period of tDCS application in speed-related motor tasks. However, it is not legitimate to claim that tDCS has no effect on trained athletes *per se*. More knowledge on neuronal processing of RTT and TT performance in trained athletes, the influence of tDCS parameters, and the effect of inter-individual differences are required in order to draw a comprehensive picture of whether tDCS can help to enhance motor abilities on a high performance level.

## Author Contributions

All experiments were conducted at the Max Planck Institute for Human Cognitive and Brain Sciences Leipzig. OS and PR designed the study and experimental set-up. Participants were recruited and tested by OS. OS analyzed the data. All authors interpreted the data, contributed to the manuscript, reviewed it, approved the final version content and agree to be accountable for all aspects of the work. All persons designated as authors qualify for authorship, and all those who qualified for authorship are listed.

## Conflict of Interest Statement

The authors declare that the research was conducted in the absence of any commercial or financial relationships that could be construed as a potential conflict of interest.

## References

[B1] Akarsu S. Caliskan E. Dane S. (2009). Athletes have faster eye-hand visual reaction times and higher scores on visuospatial intelligence than nonathletes. Turk. J. Med. Sci. 39, 871–874. 10.3906/sag-0809-44

[B2] Ando S. Kida N. Oda S. (2001). Central and peripheral visual reaction time of soccer players and nonathletes. Percept. Mot. Skills 92, 786–794. 10.2466/pms.92.3.786-794 11453206

[B3] Angius L. Mauger A. R. Hopker J. Pascual-Leone A. Santarnecchi E. Marcora S. M. (2018). Bilateral extracephalic transcranial direct current stimulation improves endurance performance in healthy individuals. Brain Stimul. 11, 108–117. 10.1016/j.brs.2017.09.017 29079458 PMC6298602

[B4] Aparício L. V. M. Guarienti F. Razza L. B. Carvalho A. F. Fregni F. Brunoni A. R. (2016). A systematic review on the acceptability and tolerability of transcranial direct current stimulation treatment in neuropsychiatry trials. Brain Stimul. 9, 671–681. 10.1016/j.brs.2016.05.004 27261431

[B5] Arias P. Robles-García V. Espinosa N. Corral Y. Cudeiro J. (2012). Validity of the finger tapping test in Parkinson’s disease, elderly and young healthy subjects: is there a role for central fatigue? Clin. Neurophysiol. 123, 2034–2041. 10.1016/j.clinph.2012.04.001 22560636

[B6] Atan T. Akyol P. (2014). Reaction times of different branch athletes and correlation between reaction time parameters. Proc. Soc. Behav. Sci. 116, 2886–2889. 10.1016/j.sbspro.2014.01.674

[B7] Banissy M. J. Muggleton N. G. (2013). Transcranial direct current stimulation in sports training: potential approaches. Front. Hum. Neurosci. 7:129. 10.3389/fnhum.2013.00129 23576976 PMC3620504

[B8] Biel A. L. Friedrich E. V. C. (2018). Why you should report bayes factors in your transcranial brain stimulation studies. Front. Psychol. 9:1125. 10.3389/fpsyg.2018.01125 30013501 PMC6036265

[B9] Boehringer A. Macher K. Dukart J. Villringer A. Pleger B. (2013). Cerebellar transcranial direct current stimulation modulates verbal working memory. Brain Stimul. 6, 649–653. 10.1016/j.brs.2012.10.001 23122917

[B10] Boggio P. S. Castro L. O. Savagim E. A. Braite R. Cruz V. C. Rocha R. R.. (2006). Enhancement of non-dominant hand motor function by anodal transcranial direct current stimulation. Neurosci. Lett. 404, 232–236. 10.1016/j.neulet.2006.05.051 16808997

[B11] Bolognini N. Pascual-Leone A. Fregni F. (2009). Using non-invasive brain stimulation to augment motor training-induced plasticity. J. Neuroeng. Rehabil. 6:8. 10.1186/1743-0003-6-8 19292910 PMC2667408

[B12] Bullitt E. Rahman F. N. Smith J. K. Kim E. Zeng D. Katz L. M.. (2009). The effect of exercise on the cerebral vasculature of healthy aged subjects as visualized by MR angiography. Am. J. Neuroradiol. 30, 1857–1863. 10.3174/ajnr.a1695 19589885 PMC7051270

[B13] Carlsen A. N. Eagles J. S. MacKinnon C. D. (2015). Transcranial direct current stimulation over the supplementary motor area modulates the preparatory activation level in the human motor system. Behav. Brain Res. 279, 68–75. 10.1016/j.bbr.2014.11.009 25446764 PMC4857713

[B14] Cogiamanian F. Marceglia S. Ardolino G. Barbieri S. Priori A. (2007). Improved isometric force endurance after transcranial direct current stimulation over the human motor cortical areas. Eur. J. Neurosci. 26, 242–249. 10.1111/j.1460-9568.2007.05633.x 17614951

[B15] Colcombe S. J. Erickson K. I. Scalf P. E. Kim J. S. Prakash R. McAuley E.. (2006). Aerobic exercise training increases brain volume in aging humans. J. Gerontol. A Biol. Sci. Med. Sci. 61, 1166–1170. 10.1093/gerona/61.11.1166 17167157

[B16] Colzato L. S. Nitsche M. A. Kibele A. (2017). Noninvasive brain stimulation and neural entrainment enhance athletic performance—a review. J. Cogn. Enhanc. 1, 73–79. 10.1007/s41465-016-0003-2

[B17] Crosson B. Sadek J. R. Maron L. Gökçay D. Mohr C. M. Auerbach E. J.. (2001). Relative shift in activity from medial to lateral frontal cortex during internally versus externally guided word generation. J. Cogn. Neurosci. 13, 272–283. 10.1162/089892901564225 11244551

[B18] Davis N. J. (2013). Neurodoping: brain stimulation as a performance-enhancing measure. Sports Med. 43, 649–653. 10.1007/s40279-013-0027-z 23504390

[B19] Davranche K. Burle B. Audiffren M. Hasbroucq T. (2006). Physical exercise facilitates motor processes in simple reaction time performance: an electromyographic analysis. Neurosci. Lett. 396, 54–56. 10.1016/j.neulet.2005.11.008 16406344

[B20] Devanathan D. Madhavan S. (2016). Effects of anodal tDCS of the lower limb M1 on ankle reaction time in young adults. Exp. Brain Res. 234, 377–385. 10.1007/s00221-015-4470-y 26487179 PMC5015891

[B21] Dienes Z. (2011). Bayesian versus orthodox statistics: which side are you on? Perspect. Psychol. Sci. 6, 274–290. 10.1177/1745691611406920 26168518

[B22] Drummond N. M. Hayduk-Costa G. Leguerrier A. Carlsen A. N. (2017). Effector-independent reduction in choice reaction time following bi-hemispheric transcranial direct current stimulation over motor cortex. PLoS One 12:e0172714. 10.1371/journal.pone.0172714 28263998 PMC5338788

[B23] Dunst B. Benedek M. Jauk E. Bergner S. Koschutnig K. Sommer M.. (2014). Neural efficiency as a function of task demands. Intelligence 42, 22–30. 10.1016/j.intell.2013.09.005 24489416 PMC3907682

[B24] Dutta A. Chugh S. Banerjee A. Dutta A. (2014). Point-of-care-testing of standing posture with Wii balance board and Microsoft Kinect during transcranial direct current stimulation: a feasibility study. NeuroRehabilitation 34, 789–798. 10.3233/NRE-141077 24784496

[B25] Edwards D. J. Cortes M. Wortman-Jutt S. Putrino D. Bikson M. Thickbroom G.. (2017). Transcranial direct current stimulation and sports performance. Front. Hum. Neurosci. 11:243. 10.3389/fnhum.2017.00243 28539880 PMC5423975

[B26] Erickson K. I. Weinstein A. M. Sutton B. P. Prakash R. S. Voss M. W. Chaddock L.. (2012). Beyond vascularization: aerobic fitness is associated with N-acetylaspartate and working memory. Brain Behav. 2, 32–41. 10.1002/brb3.30 22574272 PMC3343297

[B27] Flöel A. (2014). tDCS-enhanced motor and cognitive function in neurological diseases. Neuroimage 85, 934–947. 10.1016/j.neuroimage.2013.05.098 23727025

[B28] Furuya S. Nitsche M. A. Paulus W. Altenmüller E. (2013). Early optimization in finger dexterity of skilled pianists: implication of transcranial stimulation. BMC Neurosci. 14:35. 10.1186/1471-2202-14-35 23496918 PMC3616936

[B29] Gandevia S. C. (2001). Spinal and supraspinal factors in human muscle fatigue. Physiol. Rev. 81, 1725–1789. 10.1152/physrev.2001.81.4.1725 11581501

[B30] Gandiga P. C. Hummel F. C. Cohen L. G. (2006). Transcranial DC stimulation (tDCS): a tool for double-blind sham-controlled clinical studies in brain stimulation. Clin. Neurophysiol. 117, 845–850. 10.1016/j.clinph.2005.12.003 16427357

[B31] Geiger A. Cleeremans A. Bente G. Vogeley K. (2018). Social cues alter implicit motor learning in a serial reaction time task. Front. Hum. Neurosci. 12:197. 10.3389/fnhum.2018.00197 29867420 PMC5960666

[B32] Goldberg G. (1985). Supplementary motor area structure and function: review and hypotheses. Behav. Brain Sci. 8:567 10.1017/s0140525x00045167

[B33] Hazime F. A. da Cunha R. A. Soliaman R. R. Romancini A. C. B. Pochini A. D. C. Ejnisman B. (2017). Anodal transcranial direct current stimulation (TDCS) increases isometric strength of shoulder rotators muscles in handball players. Int. J. Sports Phys. Ther. 12, 402–407. 28593094 PMC5455189

[B34] Horvath J. C. Carter O. Forte J. D. (2016). No significant effect of transcranial direct current stimulation (tDCS) found on simple motor reaction time comparing 15 different simulation protocols. Neuropsychologia 91, 544–552. 10.1016/j.neuropsychologia.2016.09.017 27664296

[B36] Hummel F. Cohen L. G. (2005). Improvement of motor function with noninvasive cortical stimulation in a patient with chronic stroke. Neurorehabil. Neural Repair 19, 14–19. 10.1177/1545968304272698 15673839

[B35] Hummel F. C. Voller B. Celnik P. Floel A. Giraux P. Gerloff C.. (2006). Effects of brain polarization on reaction times and pinch force in chronic stroke. BMC Neurosci. 7:73. 10.1186/1471-2202-7-73 17083730 PMC1636653

[B37] Hupfeld K. E. Ketcham C. J. Schneider H. D. (2017). Transcranial direct current stimulation (tDCS) to the supplementary motor area (SMA) influences performance on motor tasks. Exp. Brain Res. 235, 851–859. 10.1007/s00221-016-4848-5 27909747

[B38] Ishigaki H. Miyao M. (1993). Differences in dynamic visual acuity between athletes and nonathletes. Percept. Mot. Skills 77, 835–839. 10.2466/pms.1993.77.3.835 8284163

[B39] Jäncke L. Koeneke S. Hoppe A. Rominger C. Hänggi J. (2009). The architecture of the golfer’s brain. PLoS One 4:e4785. 10.1371/journal.pone.0004785 19277116 PMC2650782

[B40] Jeffery D. T. Norton J. A. Roy F. D. Gorassini M. A. (2007). Effects of transcranial direct current stimulation on the excitability of the leg motor cortex. Exp. Brain Res. 182, 281–287. 10.1007/s00221-007-1093-y 17717651

[B41] Jeffreys H. (1961). The Theory of Probability, 3rd ed. Oxford Classic Texts in the Physical Sciences Oxford: Oxford University Press.

[B42] Jurcak V. Tsuzuki D. Dan I. (2007). 10/20, 10/10, and 10/5 systems revisited: their validity as relative head-surface-based positioning systems. Neuroimage 34, 1600–1611. 10.1016/j.neuroimage.2006.09.024 17207640

[B43] Kaminski E. Steele C. J. Hoff M. Gundlach C. Rjosk V. Sehm B.. (2016). Transcranial direct current stimulation (tDCS) over primary motor cortex leg area promotes dynamic balance task performance. Clin. Neurophysiol. 127, 2455–2462. 10.1016/j.clinph.2016.03.018 27178865

[B44] Kashihara K. Nakahara Y. (2005). Short-term effect of physical exercise at lactate threshold on choice reaction time. Percept. Mot. Skills 100, 275–291. 10.2466/pms.100.2.275-291 15974335

[B45] Kruschke J. K. (2011). Bayesian assessment of null values via parameter estimation and model comparison. Perspect. Psychol. Sci. 6, 299–312. 10.1177/1745691611406925 26168520

[B46] Kuan Y. M. Zuhairi N. A. Manan F. A. Knight V. F. Omar R. (2018). Visual reaction time and visual anticipation time between athletes and non-athletes. Malaysian J. Public Health Med. 1, 135–141.

[B47] Kwon Y. H. Kang K. W. Son S. M. Lee N. K. (2015). Is effect of transcranial direct current stimulation on visuomotor coordination dependent on task difficulty? Neural Regen. Res. 10, 463–466. 10.4103/1673-5374.153697 25878597 PMC4396111

[B48] Laby D. M. Kirschen D. G. Govindarajulu U. DeLand P. (2018). The hand-eye coordination of professional baseball players: the relationship to batting. Optom. Vis. Sci. 95, 557–567. 10.1097/opx.0000000000001239 29985271

[B49] Laczó B. Antal A. Rothkegel H. Paulus W. (2014). Increasing human leg motor cortex excitability by transcranial high frequency random noise stimulation. Restor. Neurol. Neurosci. 32, 403–410. 10.3233/RNN-130367 24576783

[B50] Larsson B. Månsson B. Karlberg C. Syvertsson P. Elert J. Gerdle B. (1999). Reproducibility of surface EMG variables and peak torque during three sets of ten dynamic contractions. J. Electromyogr. Kinesiol. 9, 351–357. 10.1016/s1050-6411(99)00006-1 10527216

[B51] Lattari E. Campos C. Lamego M. K. Passos de Souza S. L. Neto G. M. Rocha N. B.. (2017a). Can transcranial direct current stimulation improve muscle power in individuals with advanced resistance training experience? J. Strength Cond. Res. [Epub ahead of print]. 10.1519/jsc.0000000000001956 28426515

[B52] Lattari E. Costa S. S. Campos C. de Oliveira A. J. Machado S. Maranhao Neto G. A. (2017b). Can transcranial direct current stimulation on the dorsolateral prefrontal cortex improves balance and functional mobility in Parkinson’s disease? Neurosci. Lett. 636, 165–169. 10.1016/j.neulet.2016.11.019 27838447

[B53] Lee M. D. Wagenmakers E.-J. (2013). Bayesian Cognitive Modeling: A Practical Course. New York, NY: Cambridge University Press.

[B54] Levasseur-Moreau J. Brunelin J. Fecteau S. (2013). Non-invasive brain stimulation can induce paradoxical facilitation. Are these neuroenhancements transferable and meaningful to security services? Front. Hum. Neurosci. 7:449. 10.3389/fnhum.2013.00449 23966923 PMC3743213

[B55] Little T. Williams A. G. (2005). Specificity of acceleration, maximum speed, and agility in professional soccer players. J. Strength Cond. Res. 19, 76–78. 10.1519/00124278-200502000-00013 15705049

[B56] Lulic T. El-Sayes J. Fassett H. J. Nelson A. J. (2017). Physical activity levels determine exercise-induced changes in brain excitability. PLoS One 12:e0173672. 10.1371/journal.pone.0173672 28278300 PMC5344515

[B57] Machado D. G. D. S. Unal G. Andrade S. M. Moreira A. Altimari L. R. Brunoni A. R.. (2018). Effect of transcranial direct current stimulation on exercise performance: a systematic review and meta-analysis. Brain Stimul. [Epub ahead of print]. 10.1016/j.brs.2018.12.227 30630690

[B58] Madhavan S. Stinear J. W. (2010). Focal and bi-directional modulation of lower limb motor cortex using anodal transcranial direct current stimulation. Brain Stimul. 3:42. 10.1016/j.brs.2009.06.005 20161639 PMC2818023

[B59] Marsman M. Wagenmakers E.-J. (2017). Bayesian benefits with JASP. Eur. J. Dev. Psychol. 14, 545–555. 10.1080/17405629.2016.1259614

[B60] Martin T. Houck J. M. Bish J. P. Kicić D. Woodruff C. C. Moses S. N.. (2006). MEG reveals different contributions of somatomotor cortex and cerebellum to simple reaction time after temporally structured cues. Hum. Brain Mapp. 27, 552–561. 10.1002/hbm.20200 16247784 PMC6871412

[B61] Meier J. Topka M. S. Hänggi J. (2016). Differences in cortical representation and structural connectivity of hands and feet between professional handball players and ballet dancers. Neural Plast. 2016:6817397. 10.1155/2016/6817397 27247805 PMC4876236

[B62] Miles J. Shevlin M. (2000). Applying Regression and Correlation: A Guide for Students and Researchers. London, UK; CA, USA; New Delhi, India: Sage publications Ltd.

[B63] Mizuguchi N. Katayama T. Kanosue K. (2018). The effect of cerebellar transcranial direct current stimulation on a throwing task depends on individual level of task performance. Neuroscience 371, 119–125. 10.1016/j.neuroscience.2017.11.048 29223349

[B65] Nitsche M. A. Liebetanz D. Lang N. Antal A. Tergau F. Paulus W. (2003a). Safety criteria for transcranial direct current stimulation (tDCS) in humans. Clin. Neurophysiol. 114, 2220–2222; author reply 2222–2223. 10.1016/s1388-2457(03)00235-9 14580622

[B66] Nitsche M. A. Schauenburg A. Lang N. Liebetanz D. Exner C. Paulus W.. (2003b). Facilitation of implicit motor learning by weak transcranial direct current stimulation of the primary motor cortex in the human. J. Cogn. Neurosci. 15, 619–626. 10.1162/089892903321662994 12803972

[B64] Nitsche M. A. Paulus W. (2000). Excitability changes induced in the human motor cortex by weak transcranial direct current stimulation. J. Physiol. 527, 633–639. 10.1111/j.1469-7793.2000.t01-1-00633.x 10990547 PMC2270099

[B67] Okano A. H. Fontes E. B. Montenegro R. A. Farinatti Pde T. V. Cyrino E. S. Li L. M.. (2015). Brain stimulation modulates the autonomic nervous system, rating of perceived exertion and performance during maximal exercise. Br. J. Sports Med. 49, 1213–1218. 10.1136/bjsports-2012-091658 23446641

[B68] Oldfield R. C. (1971). The assessment and analysis of handedness: the Edinburgh inventory. Neuropsychologia 9, 97–113. 10.1016/0028-3932(71)90067-4 5146491

[B69] Pain M. T. G. Hibbs A. (2007). Sprint starts and the minimum auditory reaction time. J. Sports Sci. 25, 79–86. 10.1080/02640410600718004 17127583

[B70] Park I. S. Lee K. J. Han J. W. Lee N. J. Lee W. T. Park K. A.. (2009). Experience-dependent plasticity of cerebellar vermis in basketball players. Cerebellum 8, 334–339. 10.1007/s12311-009-0100-1 19259755

[B71] Paul M. Biswas S. K. Sandhu S. J. (2011). Role of sports vision and eye hand coordination training in performance of table tennis players. Braz. J. Biomot. 5, 106–116.

[B72] Pesta D. H. Angadi S. S. Burtscher M. Roberts C. K. (2013). The effects of caffeine, nicotine, ethanol, and tetrahydrocannabinol on exercise performance. Nutr. Metab. 10:71. 10.1186/1743-7075-10-71 24330705 PMC3878772

[B73] Ragert P. Camus M. Vandermeeren Y. Dimyan M. A. Cohen L. G. (2009). Modulation of effects of intermittent theta burst stimulation applied over primary motor cortex (M1) by conditioning stimulation of the opposite M1. J. Neurophysiol. 102, 766–773. 10.1152/jn.00274.2009 19474173 PMC2724345

[B74] Reardon S. (2016). ‘Brain doping’ may improve athletes’ performance. Nature 531, 283–284. 10.1038/nature.2016.19534 26983516

[B75] Ridding M. C. Ziemann U. (2010). Determinants of the induction of cortical plasticity by non-invasive brain stimulation in healthy subjects. J. Physiol. 588, 2291–2304. 10.1113/jphysiol.2010.190314 20478978 PMC2915507

[B76] Saimpont A. Mercier C. Malouin F. Guillot A. Collet C. Doyon J.. (2016). Anodal transcranial direct current stimulation enhances the effects of motor imagery training in a finger tapping task. Eur. J. Neurosci. 43, 113–119. 10.1111/ejn.13122 26540137

[B77] Salimpour Y. Shadmehr R. (2014). Motor costs and the coordination of the two arms. J. Neurosci. 34, 1806–1818. 10.1523/JNEUROSCI.3095-13.2014 24478362 PMC3905146

[B78] Schlaffke L. Lissek S. Lenz M. Brüne M. Juckel G. Hinrichs T.. (2014). Sports and brain morphology—a voxel-based morphometry study with endurance athletes and martial artists. Neuroscience 259, 35–42. 10.1016/j.neuroscience.2013.11.046 24291669

[B79] Seidel O. Carius D. Kenville R. Ragert P. (2017). Motor learning in a complex balance task and associated neuroplasticity: a comparison between endurance athletes and nonathletes. J. Neurophysiol. 118, 1849–1860. 10.1152/jn.00419.2017 28659467 PMC5599667

[B81] Stagg C. J. Jayaram G. Pastor D. Kincses Z. T. Matthews P. M. Johansen-Berg H. (2011). Polarity and timing-dependent effects of transcranial direct current stimulation in explicit motor learning. Neuropsychologia 49, 800–804. 10.1016/j.neuropsychologia.2011.02.009 21335013 PMC3083512

[B80] Stagg C. J. Nitsche M. A. (2011). Physiological basis of transcranial direct current stimulation. Neuroscientist 17, 37–53. 10.1177/1073858410386614 21343407

[B82] Steele C. J. Bailey J. A. Zatorre R. J. Penhune V. B. (2013). Early musical training and white-matter plasticity in the corpus callosum: evidence for a sensitive period. J. Neurosci. 33, 1282–1290. 10.1523/JNEUROSCI.3578-12.2013 23325263 PMC6704889

[B83] Tanaka S. Hanakawa T. Honda M. Watanabe K. (2009). Enhancement of pinch force in the lower leg by anodal transcranial direct current stimulation. Exp. Brain Res. 196, 459–465. 10.1007/s00221-009-1863-9 19479243 PMC2700246

[B84] Tecchio F. Zappasodi F. Assenza G. Tombini M. Vollaro S. Barbati G.. (2010). Anodal transcranial direct current stimulation enhances procedural consolidation. J. Neurophysiol. 104, 1134–1140. 10.1152/jn.00661.2009 20538777

[B85] Théoret H. Haque J. Pascual-Leone A. (2001). Increased variability of paced finger tapping accuracy following repetitive magnetic stimulation of the cerebellum in humans. Neurosci. Lett. 306, 29–32. 10.1016/s0304-3940(01)01860-2 11403950

[B86] Vargas V. Z. Baptista A. F. Pereira G. O. C. Pochini A. C. Ejnisman B. Santos M. B.. (2018). Modulation of isometric quadriceps strength in soccer players with transcranial direct current stimulation: a crossover study. J. Strength Cond. Res. 32, 1336–1341. 10.1519/jsc.0000000000001985 28489629

[B87] Verburgh L. Scherder E. J. A. van Lange P. A. M. Oosterlaan J. (2016). The key to success in elite athletes? Explicit and implicit motor learning in youth elite and non-elite soccer players. J. Sports Sci. 34, 1782–1790. 10.1080/02640414.2015.1137344 26788666

[B88] Verissimo I. S. Barradas I. M. Santos T. T. Miranda P. C. Ferreira H. A. (2016). “Effects of prefrontal anodal transcranial direct current stimulation on working-memory and reaction time,” in 2016 38th Annual International Conference of the IEEE Engineering in Medicine and Biology Society (EMBC), Orlando, FL: IEEE, 2016, 1790–1793. 10.1109/EMBC.2016.7591065 28268675

[B89] Vitor-Costa M. Okuno N. M. Bortolotti H. Bertollo M. Boggio P. S. Fregni F.. (2015). Improving cycling performance: transcranial direct current stimulation increases time to exhaustion in cycling. PLoS One 10:e0144916. 10.1371/journal.pone.0144916 26674200 PMC4687680

[B90] Vollmann H. Ragert P. Conde V. Villringer A. Classen J. Witte O. W.. (2014). Instrument specific use-dependent plasticity shapes the anatomical properties of the corpus callosum: a comparison between musicians and non-musicians. Front. Behav. Neurosci. 8:245. 10.3389/fnbeh.2014.00245 25076879 PMC4100438

[B91] Voss M. W. Prakash R. S. Erickson K. I. Basak C. Chaddock L. Kim J. S.. (2010). Plasticity of brain networks in a randomized intervention trial of exercise training in older adults. Front. Aging Neurosci. 2:32. 10.3389/fnagi.2010.00032 20890449 PMC2947936

[B92] Wessel K. Zeffiro T. Toro C. Hallett M. (1997). Self-paced versus metronome-paced finger movements. A positron emission tomography study. J. Neuroimaging. 7, 145–151. 10.1111/jon199773145 9237433

[B93] Yandell K. M. Spirduso W. W. (1981). Sex and athletic status as factors in reaction latency and movement time. Res. Q. Exerc. Sport 52, 495–504. 10.1080/02701367.1981.10607895 7330443

